# Correction: Evaluation of in silico pathogenicity prediction tools for the classification of small in-frame indels

**DOI:** 10.1186/s12920-023-01732-3

**Published:** 2023-11-16

**Authors:** S. Cannon, M. Williams, A. C. Gunning, C. F. Wright

**Affiliations:** Department of Clinical and Biomedical Sciences, Medical School, Faculty of Health and Life Sciences, Innovation, Learning and Development Building, Royal Devon and Exeter Hospital, University of Exeter, Barrack Road, Exeter, EX2 5DW UK

**Correction: BMC Med Genomics 16, 36 (2023)**

10.1186/s12920-023-01454-6

Following publication of the original article [1], the authors reported errors in additional file 2. Some of the HGVS annotations in this file were incorrect, affecting two columns in the table. These annotations were not used for variant classification or pathogenicity prediction, so would not have affected the results presented in the paper. The updated additional file is supplied in this correction article. The original article [1] has been corrected.

### Electronic supplementary material

Below is the link to the electronic supplementary material.

Additional file 2: Table S1. Inframe-indels and pathogenicity predictions from tools used in this study

## References

[1] Cannon S, Williams M, Gunning AC (2023). Evaluation of in silico pathogenicity prediction tools for the classification of small in-frame indels. BMC Med Genomics.

