# Supplementary material for: Evaluation of in silico pathogenicity prediction tools for the classification of small in-frame indels
Source: BMC Med Genomics. 2023 Feb 28;16:36. doi: 10.1186/s12920-023-01454-6 (PMC9972633; doi:10.1186/s12920-023-01454-6)
Supplement: Supplementary file 1 — Additional file 1: Fig. S1. ROC curves and AUCs using (A) 3964 in-frame indels from gnomAD, ClinVar and the DDD study (B) 151 in-frame indels only observed in the DDD study. Only tools producing continuous data as output are plotted (n=7). Fig. S2. Performance of nine pathogenicity prediction tools versus increasing variant length in a dataset of 3964 in-frame indels aggregated from gnomAD, ClinVar and the DDD study. Fig. S3. Sensitivity and specificity of 9 pathogenicity prediction algorithms with increasing variant length in a dataset of in-frame (A) deletions (n=2718) and (B) insertions (n=1246). [file 12920_2023_1454_MOESM1_ESM.docx]

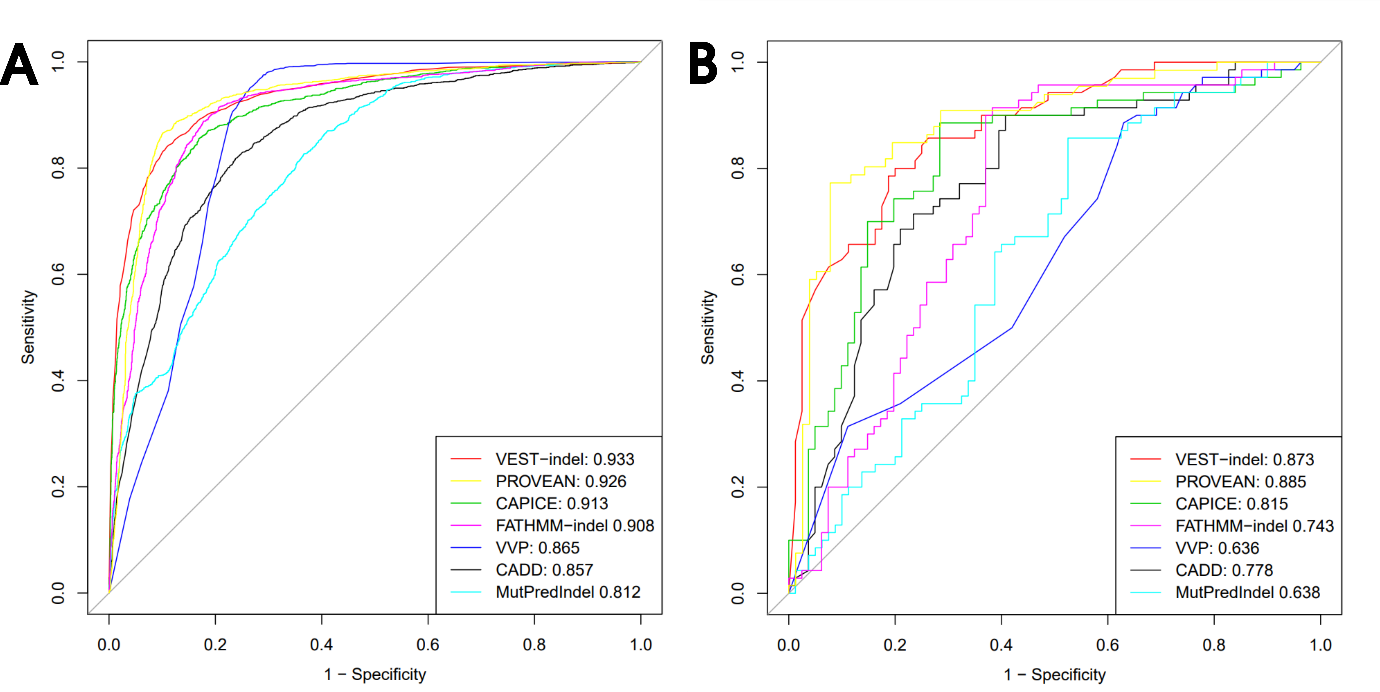
**Figure S1**. ROC curves and AUCs using **(A)** 3964 in-frame indels from gnomAD, ClinVar and the DDD study **(B)** 151 in-frame indels only observed in the DDD study. Only tools producing continuous data as output are plotted (n=7).


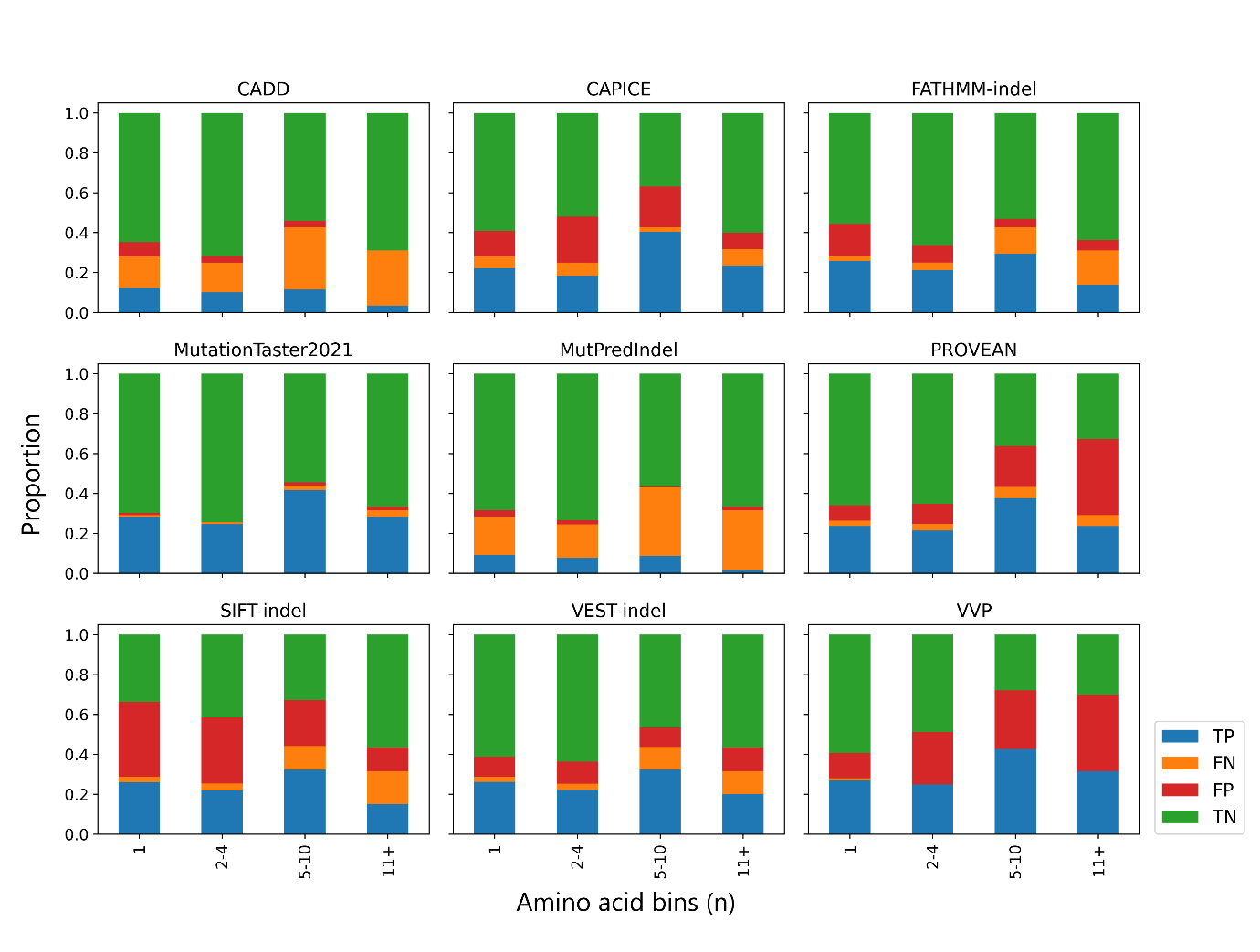
**Figure S2**. Performance of nine pathogenicity prediction tools versus increasing variant length in a dataset of 3964 in-frame indels aggregated from gnomAD, ClinVar and the DDD study.


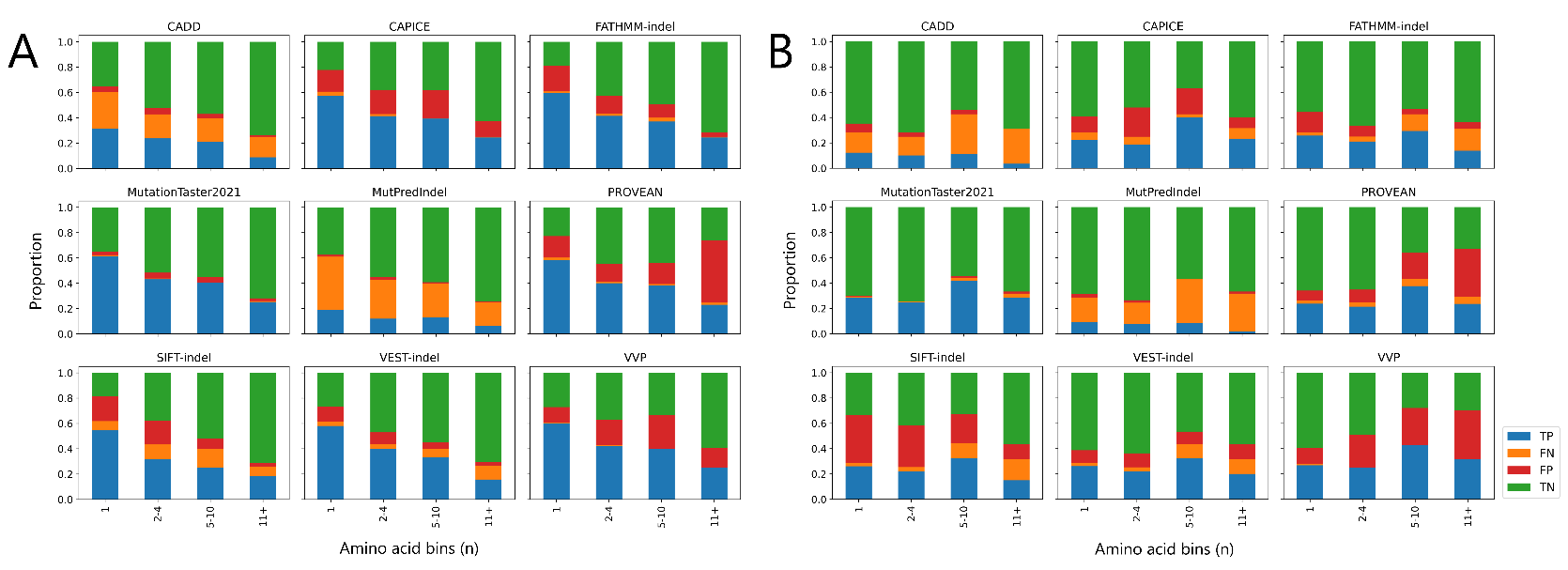
**Figure S3**. Sensitivity and specificity of 9 pathogenicity prediction algorithms with increasing variant length in a dataset of in-frame **(A)** deletions (n=2718) and **(B)** insertions (n=1246)
